# An enhanced triple fluorescence flow-cytometry-based assay shows differential activation of the Notch signaling pathway by human papillomavirus E6 proteins

**DOI:** 10.1038/s41598-022-06922-0

**Published:** 2022-02-22

**Authors:** JiaWen Lim, Elke Straub, Frank Stubenrauch, Thomas Iftner, Michael Schindler, Claudia Simon

**Affiliations:** Institute of Medical Virology and Epidemiology of Viral Diseases, University Hospital Tuebingen, Tuebingen, Germany

**Keywords:** Cell signalling, Flow cytometry, Oncogenes

## Abstract

Human papillomaviruses are DNA tumor viruses. A persistent infection with high-risk HPV types is the necessary risk factor for the development of anogenital carcinoma. The E6 protein is a viral oncoprotein that directly interacts with different cellular regulatory proteins mainly affecting the cell cycle, cellular differentiation and polarization of epithelial cells. In dependency of the phylogenetic classification of HPV different interaction partners of E6 have been described. The Notch pathway seems to be one common target of HPV, which can be up or down regulated by different E6 proteins. Our novel triple fluorescence flow-cytometry-based assay allows a semi-quantitative comparison of the E6 proteins´ effect on the Notch pathway using a Notch-responsive reporter plasmid. As a result, all E6 proteins of beta-HPV repressed the Notch reporter expression, of which HPV38 E6 showed the greatest repression potential. In contrast, alpha-HPV E6 of HPV16, activates the reporter expression most significantly, whereas E6 of HPV31 and low-risk HPV6b showed significant activation only in a p53-null cell line. Interestingly, HPV18 E6, with the second highest carcinogenic risk, shows no effect. This high divergence within different genus of HPV is important for targeting the Notch pathway regarding a potential HPV therapy.

## Introduction

Promotor activity assays are indispensable to study the regulation of gene expression. Commonly, the gene of interest is replaced by a reporter gene, whose expression is then under the control of regulatory region of the original gene of interest resulting in a reporter plasmid. Reporter genes are usually enzymes such as luciferases for bioluminescence, beta-galactosidases or beta-glucuronidase for absorbance or fluorescence; or fluorescent proteins (FP). Commonly, dual reporter systems, such as dual luciferase assay, dual FPs or combinations are applied in order to account for promotor activity and variations in transfection efficiency. Cell lysis is required in most dual luciferase-based assays prior to measurement, i.e. lysis efficiency is a further variable in these assays. Compared with luciferase enzymes, FPs do not require co-factors or substrates for their fluorescence activity and can be monitored in living cells. On the other hand, modulatory proteins, which affect the expression of the gene of interest, are co-transfected with the reporter plasmid. However, the expression level of modulatory proteins can differ from experiment to experiment and between different modulatory proteins. With flow-cytometry, the expression level of the modulatory protein genetically fused to a specific fluorescent protein could be easily identified and examined with an appropriate laser. The application of flow-cytometry combined with FPs allows to monitor single cells, excludes dead cells as well as non-transfected cells and can significantly lower the background.

Proteins are expressed at different amounts and have different turnover rates in cells meaning that just transfecting the same amount of DNA does not result in same protein levels of different proteins or protein variants. In order to compare the effect of different modulatory proteins on reporter gene expression, a quantitative assessment of the amount of modulatory protein is necessary. Concomitant western blot analysis can determine the protein amount of modulatory proteins. This analysis is offline to the promotor activity measurement, necessitates Western Blot validations of each target for quantitative analysis and is time consuming. Further, low co-transfection efficiency of plasmid DNA or low expression yields and high turnover rates of the modulatory proteins can deteriorate the signal-to-noise ratio of the reporter assay because of e.g. the high number of cells negative for the modulatory protein. To reduce this background, flow-cytometry is feasible to identify cells, which express the modulatory protein genetically fused to FPs, and monitor the reporter activity only in these positive cells.

**Figure 1:**
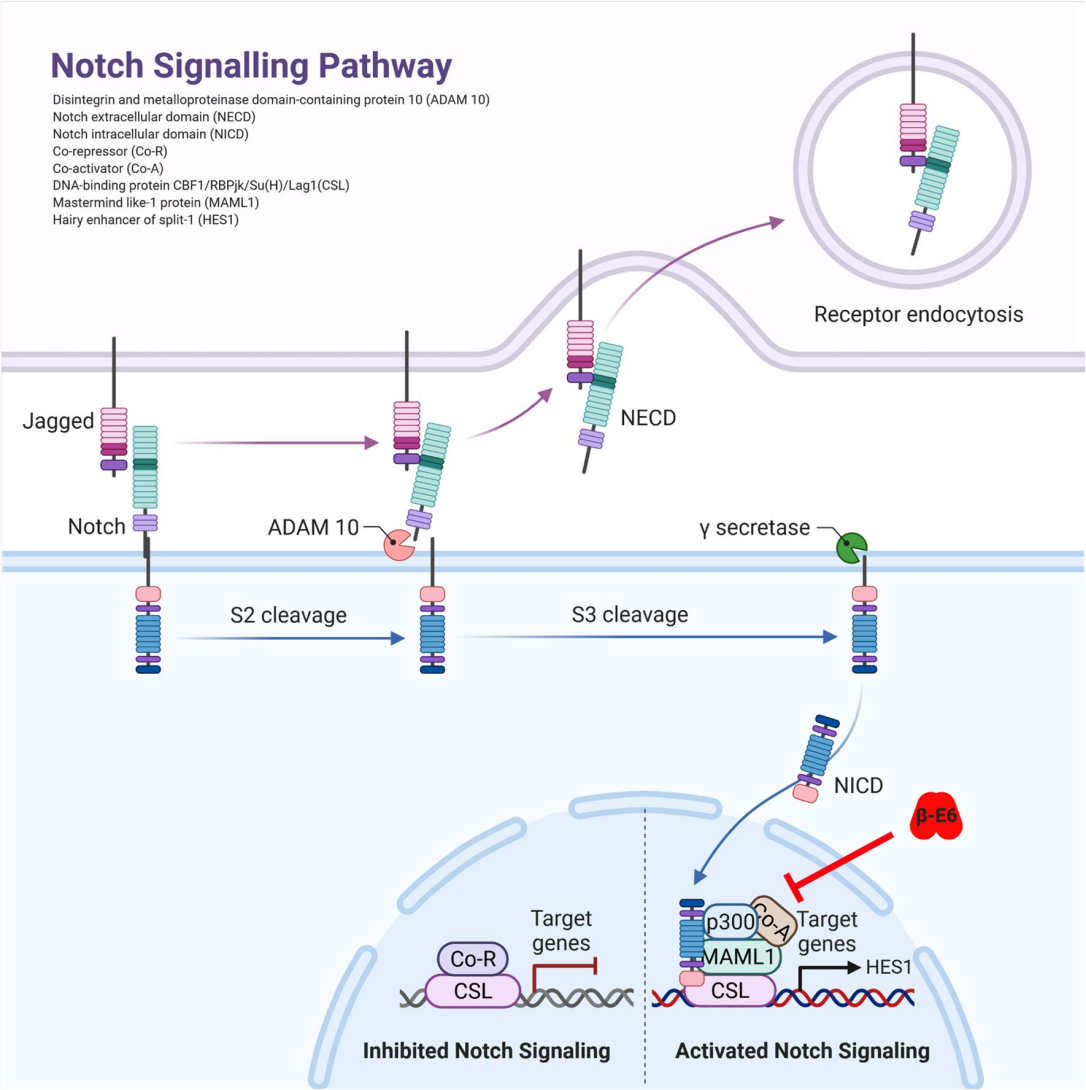
Scheme of the Notch signaling pathway. The Notch receptor is cleaved at S2 cleavage site by ADAM 10 protease upon binding to Notch ligand, Jagged from neighboring cells. Thus, releasing Notch extracellular domain (NECD) for receptor endocytosis. Cleavage of Notch intracellular domain (NICD) at S3 cleavage site by γ secretase leads to the translocation of NICD into nucleus. NICD then recruits MAML1, p300, CSL and other co-activators to activate the expression of Notch target gene, *HES1*. Beta HPV E6 protein associates with MAML1 and inhibits Notch Signaling. Adapted from “Notch Signaling Pathway”, by BioRender.com (2020). Retrieved from https://app.biorender.com/biorender-templates.

The highly conserved Notch signaling pathway plays an important role in cell proliferation and differentiation^1^. Miss-signaling is associated with cancer progression^3^, several diseases as e.g. multiple sclerosis^4–6^, as well as viral infections^7–10^. Therefore, the Notch signaling pathway is considered as a potential drug target to treat associated diseases. The signaling is triggered by direct cell-to-cell contact (Fig. 1). The activated Notch-receptor undergoes proteolytic cleavage resulting in the release of the Notch intracellular domain (NICD) to the nucleus^11^. The NICD binds to other cellular proteins (MAML1, p300, Co-Activator) recruiting the initiation complex of e.g. *HES1* gene transcription. The effect of modulatory factors such as drugs, viral or cellular proteins on the Notch signaling pathway can be monitored by measuring the *HES*1 promotor activity (*P-HES1*). In cell culture, Notch signaling pathway can be triggered by either co-culturing jagged cells, expressing the jagged protein as a Notch-receptor ligand on the cell surface (Fig. 1), or co-transfecting the NICD as an exogenous activator^12^. The second approach, however, does not allow monitoring effects upstream of NICD cleavage by gamma-secretase^13^. There are over 200 types of human papillomaviruses (HPV), of which only some repress the Notch pathway. Types of the beta genus interfere with Notch signaling via the interaction of the viral protein E6 with proteins of the transcription initiation complex for Notch-responsive expression as of *HES1*^12,14–16^. In contrast, it has been reported, that HPV16 found in 50% of all cervical cancer^17^, shows an upregulation of HES1 and Notch in cervical cancer cells^3,18–20^. This indicates, that E6 proteins of different HPV types can either up- or downregulate the Notch signaling pathway. E6 proteins of different HPV types are conserved in their amino acid sequence and structure. However, it has been shown, that the E6 proteins of different HPVs differ in their intracellular level^21^. A comparison of the modulatory effect of the individual E6 proteins on the Notch signaling necessitates information about its intracellular protein amount on a single-cell level. However, this cannot be achieved by commonly employed dual luciferase-based assays measuring luciferase expression controlled by the Notch-responsive *HES1-promotor.*

**Figure 2:**
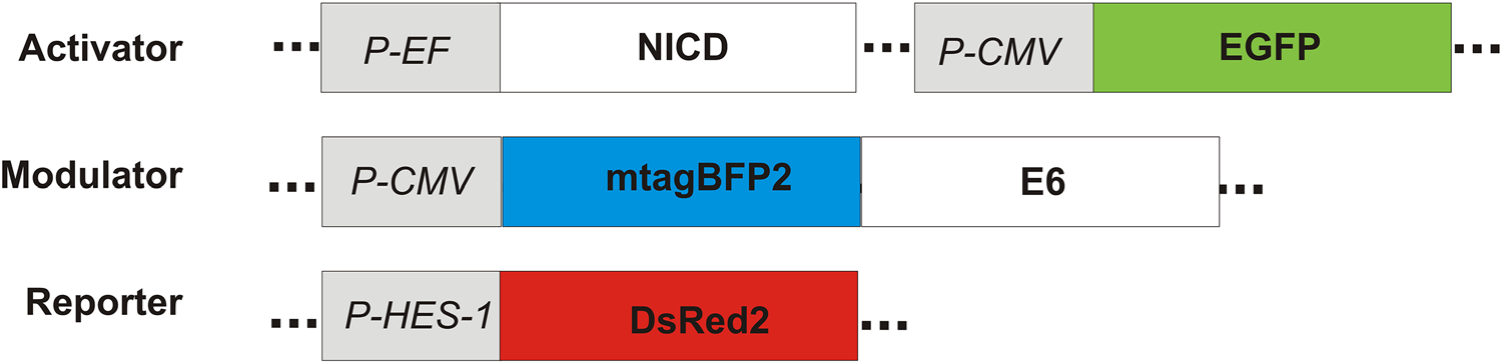
Constructs of triple fluorescence flow-cytometry-based assay to analyze *P-HES1* activity. Three plasmids are co-transfected: (I) activator plasmid with two open reading frames encoding the Notch intracellular domain (NICD) as the activator of the Notch pathway and the EGFP as transfection control for the activator plasmid. (II) the modulator plasmid, encoding the modulator protein of interest E6, which is N-terminally fused to mTagBFP2 to monitor E6 expression. (III) the reporter plasmid encoding for the reporter DsRed2. The expression of DsRed2 is under control of the *HES1* promotor which is regulated by the Notch pathway. Picture was created with CorelDRAW X7, version 17.5.0.907.

**Figure 3:**
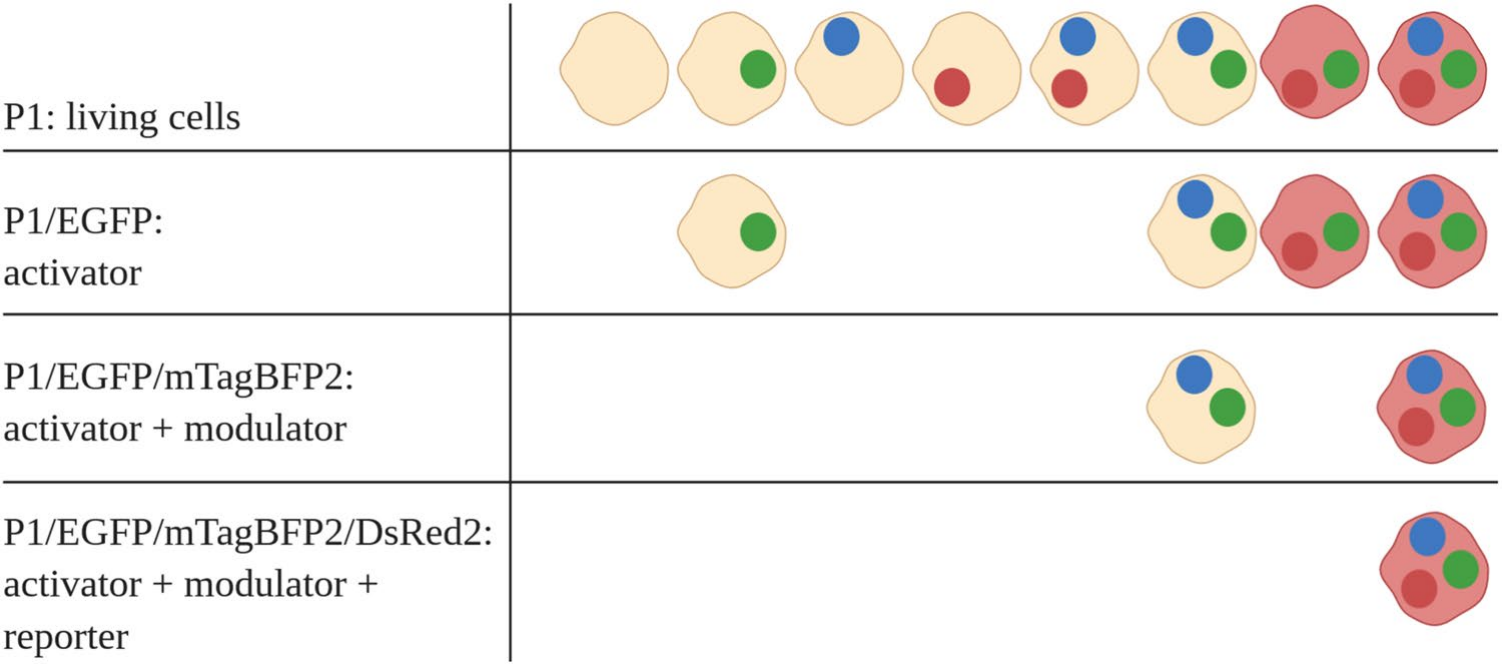
Schematic triple gating strategy. Living cells are first identified by FSC/SSC gating followed by specific inclusions of cells that are transfected with the activator plasmid (EGFP + cells). Next step is to gate on EGFP/mTagBFP2 to only monitor cells that have been transfected with the activator and the modulator (EGFP + /mTagBFP2 + cells). Then, Notch-activation is assessed in this population by measuring the DsRed2 expression (EGFP + /mTagBFP2 + /DsRed2 + cells). Picture was created with BioRender.com.

To overcome this, we developed a flow-cytometry-based assay to analyze *P-HES1* activity using three different fluorescent proteins reporting (I) the transfection of the activator plasmid, human Notch 1 intracellular domain by EGFP co-expression (here NICD), (II) the intracellular amount of the modulator (here E6) by N-terminal fusion of mTagBFP2 and (III) the promotor activity (here of *P-HES1*) by DsRed2 expression (Fig. 2). This allows us to monitor *HES1-*activation on a single cell level and consider only cells which are triple positive. By this, we can reduce the background and, additionally account for different protein expression levels (Fig. 3). Using this strategy, we assess the activating or repressing potential of E6 proteins of different genera, species and types of HPV (Table 1) on the Notch signaling pathway with a semi-quantitative approach.

**Table 1:** Tested E6 proteins of different HPV types.

Genus	Alpha				Beta		
Species	7	9		10	1		2
Tested types	18	16	31	6	5	8	38

## Results

### Exogenous activation of Notch signaling pathway in C33A and H1299 cells

**Figure 4:**
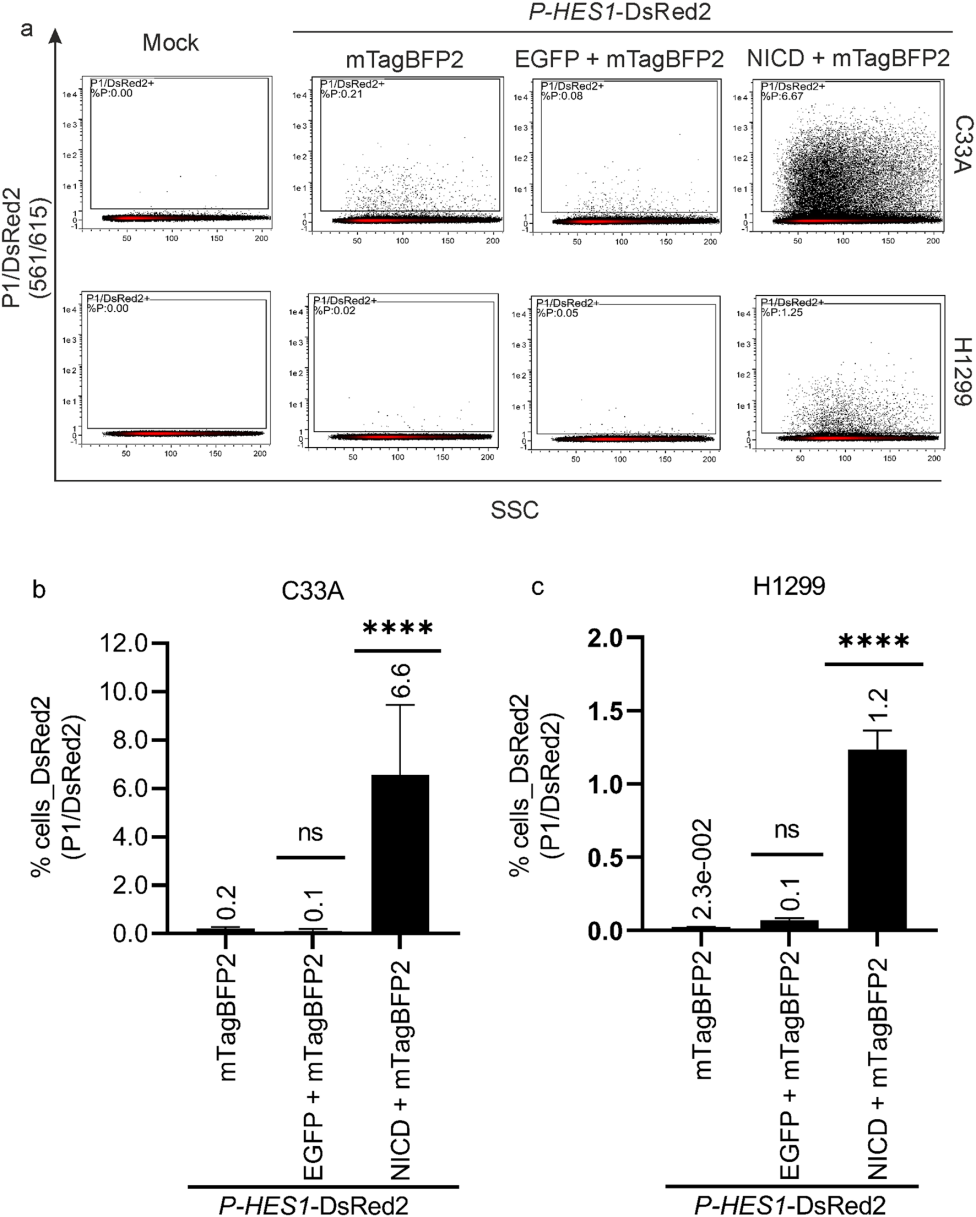
Notch activation by exogenous NICD. C33A and H1299 cells were co-transfected with 250 ng *P-HES1*-DsRed2 reporter plasmid, 250 ng EGFP or 250 ng NICD activator plasmid and 125 ng mTagBFP2 plasmid as indicated. C33A and H1299 cells were harvested for FACS measurement 48 h or 36 h post transfections, respectively. Living cells (P1) were gated for DsRed2 signal reporting the Notch pathway activity. (**a**)**.** Representative FACS plots show that the endogenous NICD is unable to activate the expression of the Notch target *HES1* gene (Column2 and Column3) sufficiently. Upon co-transfection of NICD (Column4), the increased cell population of DsRed2 positive cells clearly indicates the exogenous activation of Notch signaling. (**b, c**)**.** Three independent biological replicates were conducted. A significant cell population of DsRed2 positive cells was detected in C33A (b) and H1299 (c), respectively in presence of exogenous NICD (*P-HES1*-DsRed2 + mTagBFP2 + NICD). In contrast, the endogenous NICD does not activate *P-HES1*-DsRed2 expression sufficiently (*P-HES1*-DsRed2 + mTagBFP2), independent of the co-transfection of EGFP control plasmid (*P-HES1*-DsRed2 + mTagBFP2 + EGFP). The mean % cells of DsRed2 expressing cell populations as gated for each sample is stated above each bar and the error bars plotted indicate the standard deviation of the mean from the three independent biological replicates. P-values were calculated using One-Way ANOVA with Fischer’s LSD test by comparing the mean of each sample with mean of *P-HES1*-DsRed2 + mTagBFP2. **** = *P* ≤ 0.0001, ns = *P* > 0.05.

To verify, that the cell systems activate Notch signaling in response to exogenous NICD, we co-transfected the reporter plasmid and the control plasmid encoding for mTagBFP2 further with and without the activator plasmid and gated for DsRed2 positive cells (Fig. 4). Without the activator plasmid, C33A and H1299 cells showed less than 0.2 ± 0.06% and 0.02 ± 0.003% DsRed2 expressing cell population, respectively (Fig. 4), indicating that the constitutive and endogenous Notch signaling is low and thereby not triggering *P-HES1* regulated reporter expression sufficiently. Additionally, the co-expression of neither mTagBFP2 nor EGFP affected the signal of DsRed2. Conversely, C33A and H1299 cells co-transfected with the activator plasmid showed a significant increase in the DsRed2 expressing cell population, which was at least tenfold above the background signal of DsRed2 expressing cell population without exogenous NICD. Hence, both cell lines clearly show an activation of the Notch pathway by exogenous NICD with very low background of endogenous *P-HES1*-activity (Fig. 4b and c).

### Triple gating strategy- proof of concept

**Figure 5:**
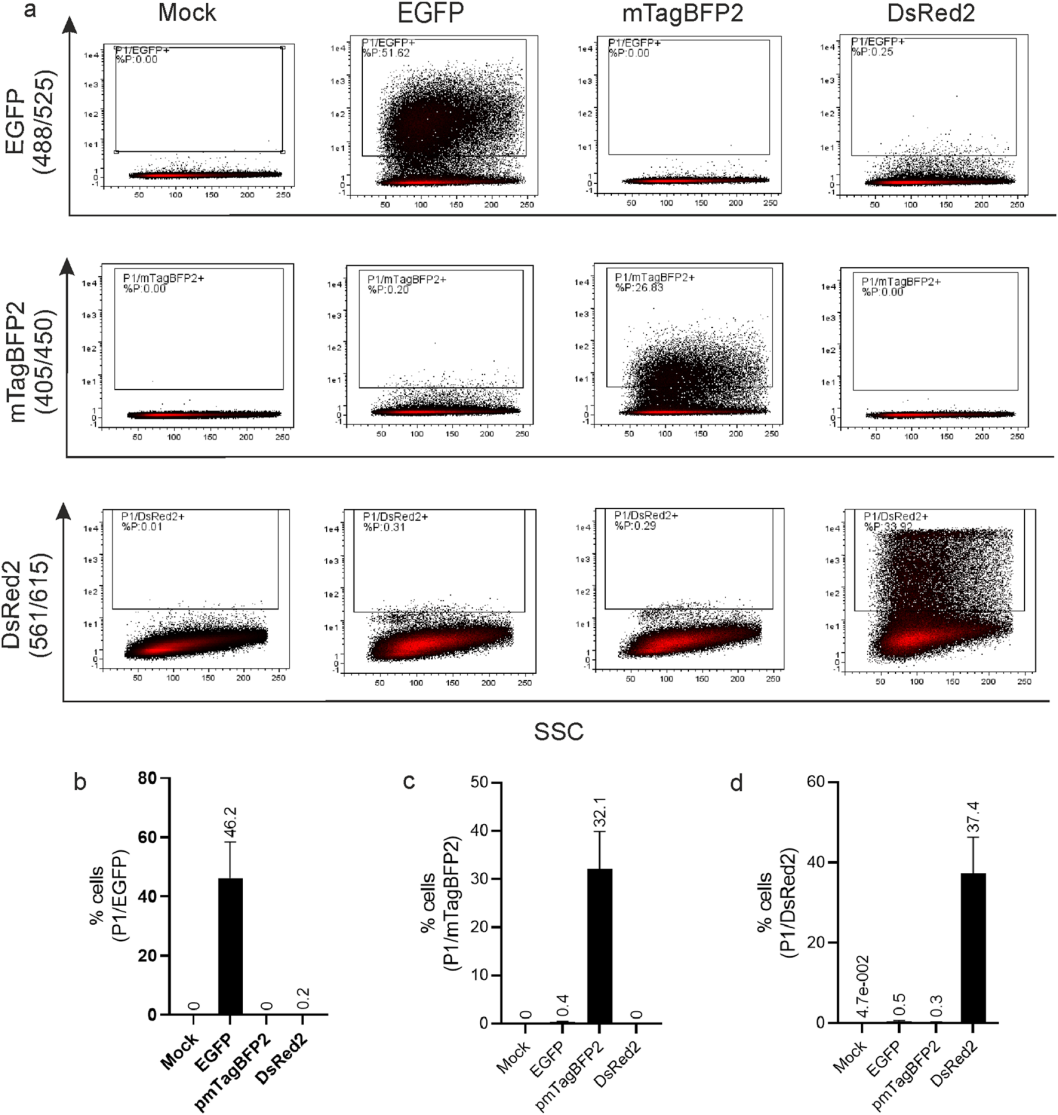
Cross talk of fluorescent proteins. (**a).** Representative FACS plots showing the crosstalk level of the three fluorescence proteins after compensation. C33A cells were transfected with control plasmid 250 ng EGFP, 125 ng mTagBFP2 or 125 ng DsRed2 individually. FACS measurement was carried out 48 h post transfection. Living cells (P1) were gated for EGFP, mTagBFP2 and DsRed2 separately to monitor the crosstalk of each fluorescence proteins in respective channel showing cell populations in % cells. EGFP showed a compensable crosstalk signal in the channel of mTagBFP2 and DsRed2. Crosstalk levels were always kept below 0.5% of cell populations in each experiment. (**b, c, d**)**.** The experiment was repeated three times. The mean % cells of cell populations as gated of three independent biological experiments are plotted in b, c and d with the mean value stated above each bar. The error bars plotted are indicating the standard deviation of mean value from the three independent biological replicates.

As the excitation and emission spectra of fluorescent proteins EGFP, mTagBFP2, DsRed2 overlap partially, it is important to carefully control for crosstalk in the three applied channels and compensate accordingly. As a control, we transfected cells individually with plasmids each encoding for EGFP, mTagBFP2 or DsRed2. Gates were set for each protein such as there is no crosstalk (< 0.5%) in the other channels (Fig. 5). By this, we were able to gate specifically for triple positive viable cells (P1/EGFP/mTagBFP2/DsRed2) showing EGFP (activator transfected), mTagBFP2 (modulator protein) and DsRed2 (Notch-responsive reporter) expression.

It had been shown previously that Notch signaling is activated when the NICD translocate into the nucleus and forms a Notch initiation complex with other cellular proteins. However, E6 proteins localize into the nucleus to interfere with formation of the Notch initiation complex. Thus, it would be interesting to know if mTagBFP2-E6s fusion retain ability to localize into the nucleus. Hence, we first analyze the mTagBFP2-E6s fusion constructs, of which all accumulated in the nucleus (Supplementary information SI1 – SI3).

**Figure 6:**
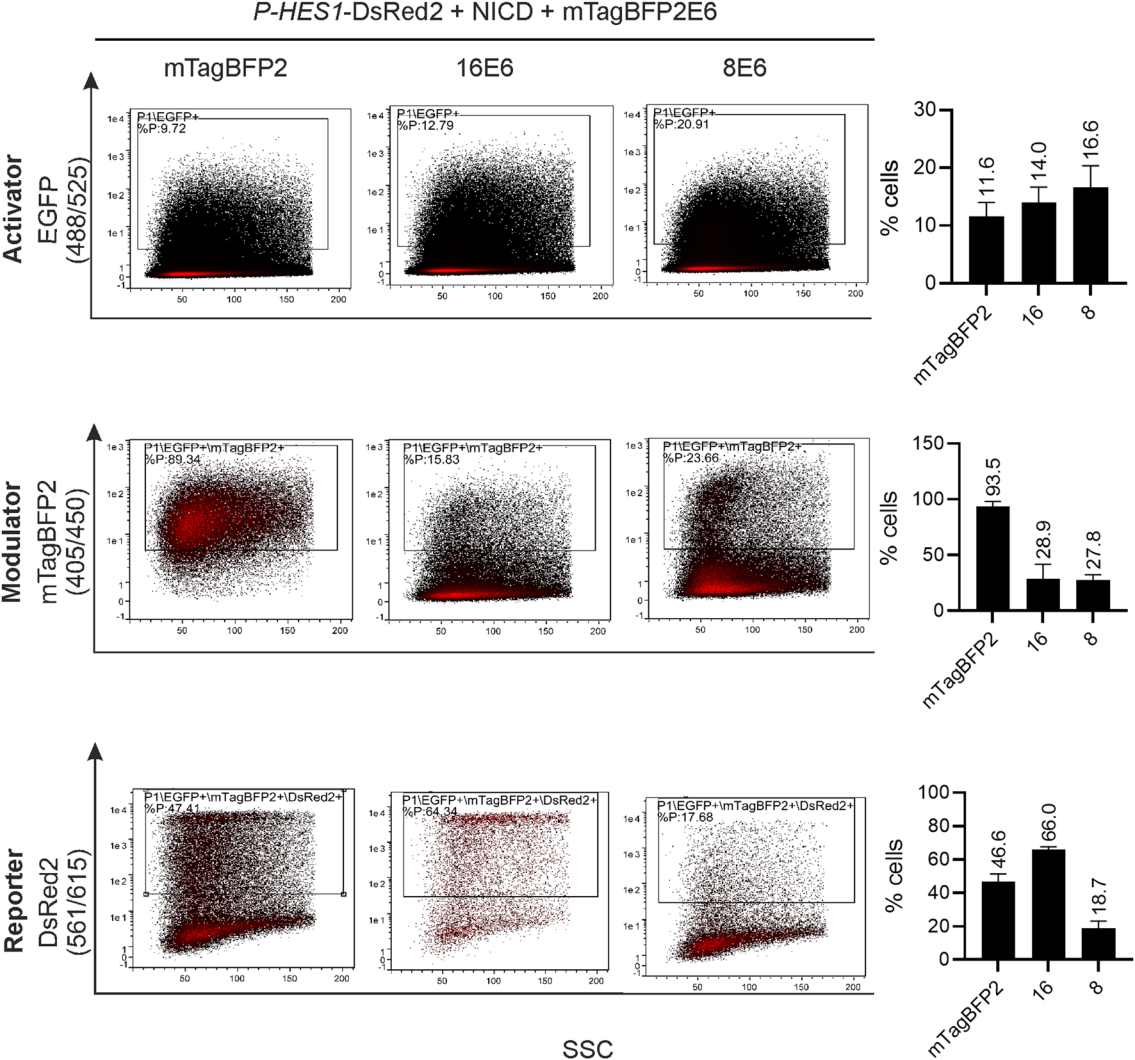
Experimental triple gating strategy, exemplary for Notch activation (16E6) and repression (8E6). C33A cells were co-transfected with 250 ng *P-HES1*-DsRed2 reporter plasmid, 250 ng NICD activator plasmid, 500 ng mTagBFP2-E6 modulator plasmid respectively or 125 ng of mTagBFP2 as a control. Cells were harvested for FACS measurement 48 h post transfection. Living cells were gated for P1/EGFP > P1/EGFP/mTagBFP2 > P1/EGFP/mTagBFP2/DsRed2 to monitor the activity of *P-HES1*. The mean % cells of cell population as gated for three independent biological replicates were plotted to the right of the representative FACS plots. The mean values were stated above each bar and the error bars indicate the standard deviation of the mean value from the three independent biological replicates.

To examine the *P-HES1* reporter activity with our triple gating strategy, an exemplary triple gating is shown for C33A co-transfected with *P-HES1* reporter plasmid, NICD activator plasmid and mTagBFP2-E6 modulator plasmid for 16E6 and 8E6 (Fig. 6). Finally, ~ 66.0 ± 1.6% cells for 16E6 and ~ 18.7 ± 4.5% cells for the E6 of the HPV8 (8E6) were DsRed2 positive in the triple gated cell population. Compared with our control (46.6 ± 4.7% cells), where we co-transfected mTagBFP2 instead of the fusion construct mTagBFP2-E6, 16E6 clearly shows an activation, while 8E6 clearly shows a repression of the *P-HES1* activity.

**Figure 7:**
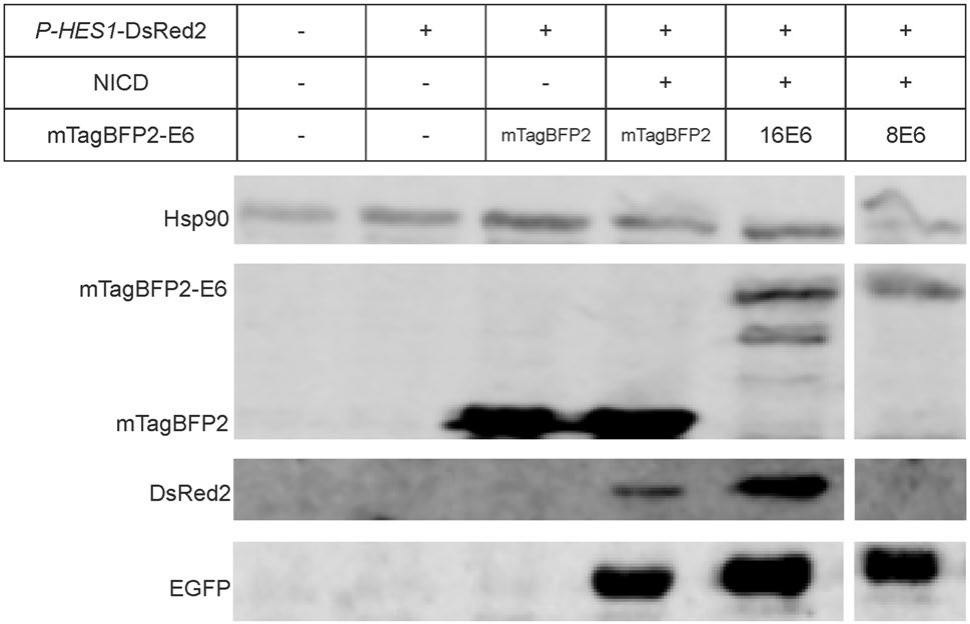
Expression of proteins by Western blot analysis. Expression level of mTagBFP2-E6, DsRed2, and EGFP were assessed by loading 70 µg of total proteins on reducing 8–20% SDS-PAGE gel. HSP90 serves as a loading control. DsRed2 protein signal is increased for 16E6 but absent for 8E6. In line with the FACS assay, these results indicate an activation by 16E6 and a repression for 8E6 of the Notch pathway. Please find full blot and detailed blot information in Figure SI4.

In parallel, we validated the results by Western Blot analysis (Fig. 7). This shows, that EGFP, mTagBFP2, mTagBFP2-E6 and DsRed2 are expressed. In accordance with the flow cytometric analyses, the *P-HES1* regulated expression of DsRed2 is activated by co-transfection of the activator plasmid (NICD), decreased for 8E6 and increased for 16E6.

### Modulation of Notch signaling pathway by different HPV E6 proteins

**Figure 8:**
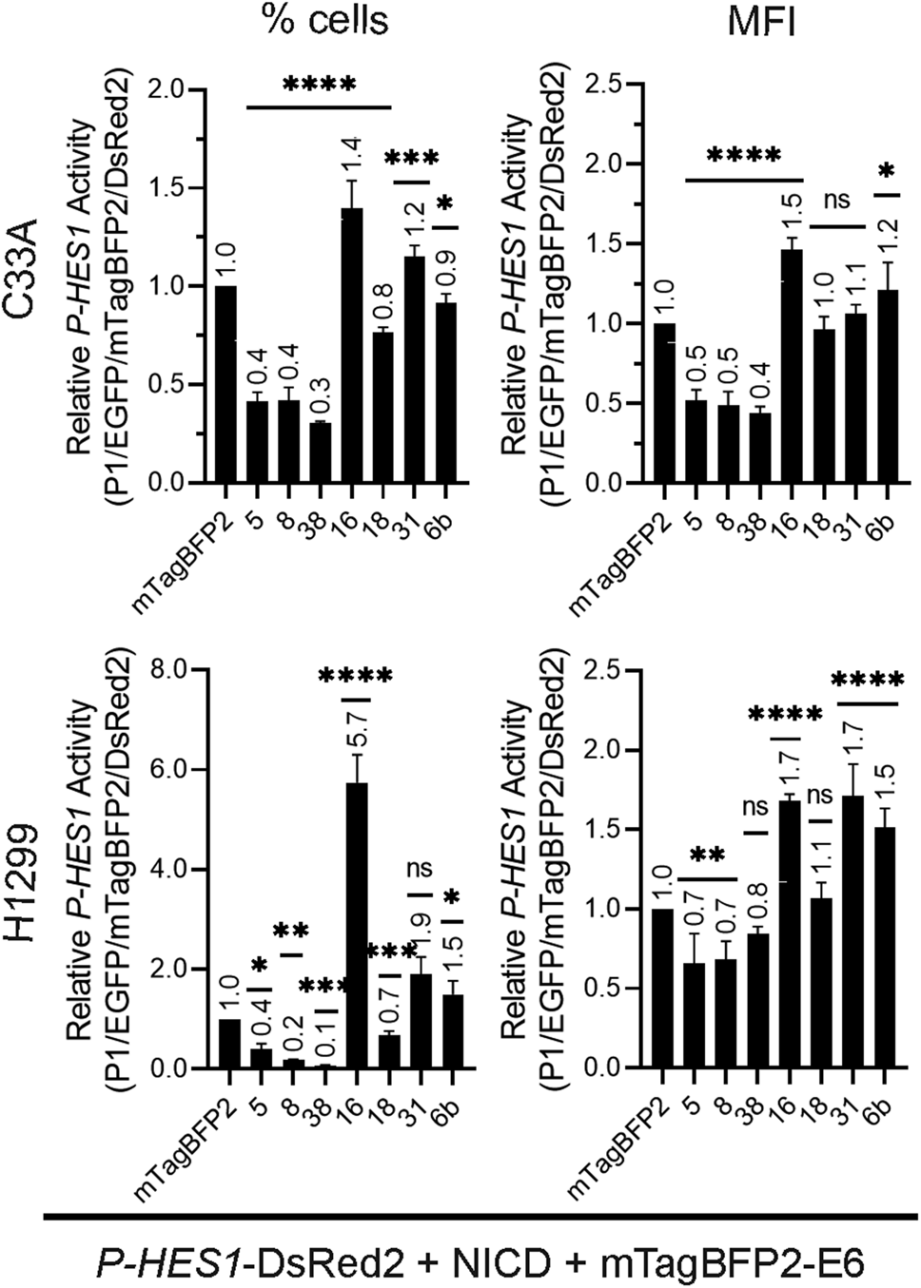
HPVE6 influence on *P-HES1* promotor activity. The influence on *P-HES1* promotor activity was analyzed by the triple gating strategy and signals for % cells (left) and mean fluorescence intensities of DsRed2 per cell (MFI, right) were plotted. Cell were co-transfected with 250 ng *P-HES1*-DsRed2 reporter plasmid, 250 ng activator NICD plasmid and 125 ng mTagBFP2 or 500 ng of respective mTagBFP2-E6. FACS measurement was carried out 48 h or 36 h post transfection for C33A and H1299 cells, respectively. Compared to the negative control (mTagBFP2 not fused to E6), E6 of beta HPV types 5, 8 and 38 inhibit DsRed2 expression in both cell lines. E6 of the alpha HPV type 16 clearly upregulates expression of DsRed2 in both cell lines. E6 of the alpha-HPV31 and 6 show a slight activation in C33A and a more pronounced activation in p53-null cell line H1299. 18E6 shows no significant effect, rather slight repressing effect in % cells in both cell lines. Data plotted are the mean values of three independent biological replicates. The number above bars are the respective mean value. The error bars plotted are the standard deviation of the mean value from three independent biological replicates. P-values were calculated using One-Way ANOVA with Fischer’s LSD test by comparing the mean of each activation and repression sample with the control activation sample from three independent experiments where * = *P* ≤ 0.05, ** = *P* ≤ 0.01, *** = *P* ≤ 0.005, **** = *P* ≤ 0.0001, ns = *P* > 0.05.

We analyzed the effect on the Notch signaling pathway of seven different E6 proteins derived from HPVs of different genus, species and carcinogenic potential (Table 1). E6 proteins interfere with p53, especially HPV types of high-risk directly degrade p53^21^. Since there is a crosstalk between p53 and the Notch pathway, we performed the same experiments in two different cell lines: the HPV-negative cervix carcinoma cell line C33A and the p53 null lung cancer cell line H1299. Applying our triple gating strategy, we analyzed the data considering the percentage of DsRed2 positive cells and the mean fluorescence intensity (MFI) (Fig. 8). Assessing MFI considers the intracellular expression level of DsRed2 produced upon activation of *P-HES1*. Analyzing the % of DsRed2 positive cells does not account for the individual single-cell level of DsRed2 expression, but rather focuses on changes on cell population levels. Nevertheless, both, % cells and MFI should change accordingly in case of repression or activation.

The E6 proteins of beta HPV 5, 8 and 38 showed a significant repression of DsRed2 + % cells and MFI in C33A and H1299 cells. In H1299 cells, repression was so potent, that the total cell numbers in the final triple-gated DsRed2 + population was below 500 cells. In contrast, the alpha-7 HPV 16E6 showed a highly significant activation of reporter expression in both cell lines.

The E6 of alpha-7 HPV 31 and alpha-10 HPV 6b show a low activation of *P-HES1* activity in C33A cells, in which activation by HPV 31 is not significant for the MFI of the DsRed2. In the p53-null H1299 cells the activation of Notch pathway by 31E6 and 6bE6 seems increased, especially for 31E6. Regarding the effect of alpha-7 HPV 18E6, in % cell-signal 18E6 shows a slight repression, whereas this effect was less clear and not significant for the MFI of the DsRed2 in both cell lines. Altogether, our triple gating strategy is suitable to monitor the modulation of the Notch signaling pathway by different E6, which show repression for all beta-HPV E6, activation for HPV16, cell-line dependent activation for HPV31 and 6b and rather no effect for HPV18.

### Semi-quantitative analysis of E6 activities

**Figure 9:**
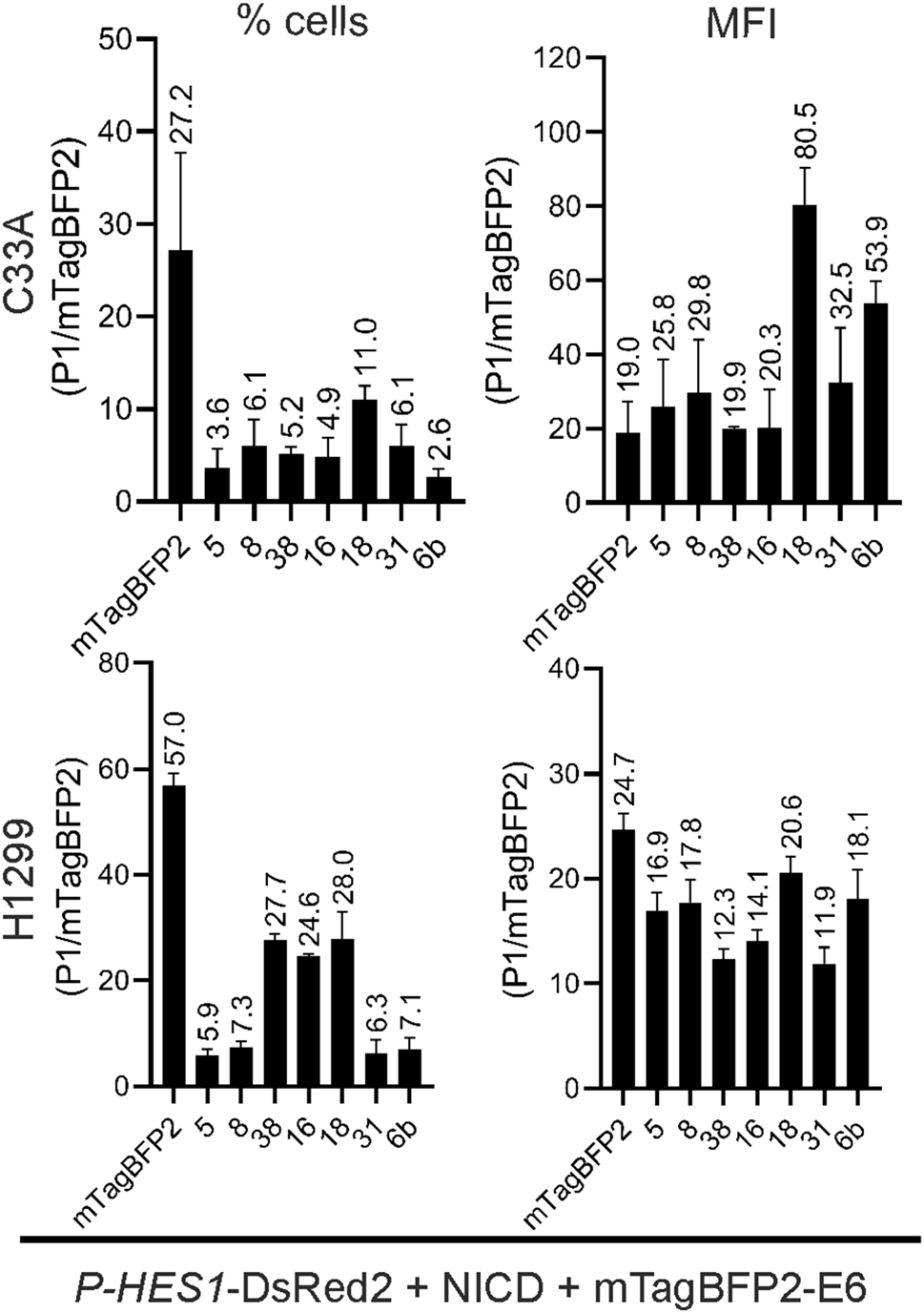
Different expression levels of mTagBFP2-E6. E6 proteins are expressed in different amounts. Cells were co-transfected with 250 ng *P-HES1*-DsRed2 reporter plasmid, 250 ng activator NICD plasmid and 125 ng mTagBFP2 or 500 ng of respective mTagBFP2-E6. FACS measurement was carried out 48 h or 36 h post transfection for C33A (top) and H1299 (lower) cells, respectively. Living cells transfected with activator, modulator and reporter plasmid were gated for mTagBFP2 signal (P1/mTagBFP2) with respect to % cells (change in population, left) and their mean fluorescence intensity per cell (intracellular level, right). Both values vary between different E6 proteins indicating that different intracellular amounts of E6 were expressed in different number of cells. Data are derived from the mean value of three independent biological replicates with the mean value stated above each bar. The error bars plotted represent the standard deviation of the mean value from the three independent biological replicates.

Both signals of mTagBFP2-E6 for % cells and MFI, , vary between the different E6 proteins (Fig. 9) indicating that the intracellular protein amount of the mTagBFP2-E6 variants differs between the different HPV types. Principally, the MFI per cell of a FP is equivalent to the amount of the respective FP per cell. Thereby, the MFI of mTagBFP2 equals to the amount of the modulator mTagBFP2-E6 and the MFI of DsRed2 equals the amount of DsRed2 as the reporter of *P-HES1* activity.

**Figure 10:**
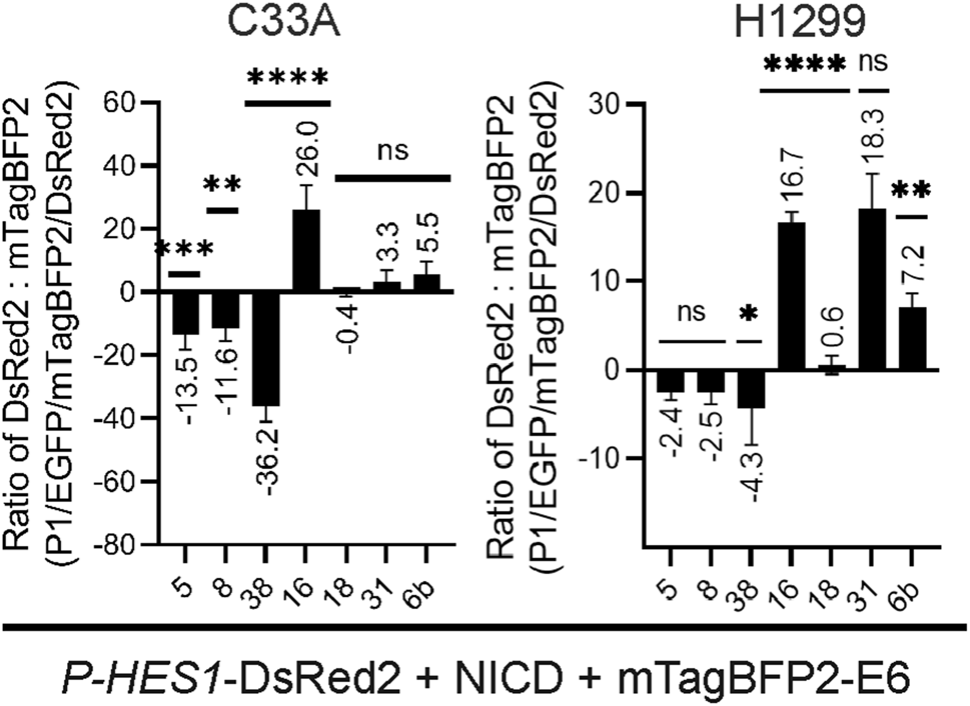
E6 activity on *P-HES1* regulated genes. The ratio of mean fluorescence intensity (MFI) of DsRed2 and mTagBFP2 were calculated for each mTagBFP2-E6 based on the triple gating strategy as described in Fig. 6 and normalized to the control mTagBFP2, which has no effect on *P-HES1* controlled DsRed2 expression (ratio set to zero). The more negative or positive the value the higher the repressing or activating activity of the respective E6. Data are derived from the average of three independent biological replicates with the mean value labelled above each bar. The error bars plotted are the standard deviation of the mean from the three independent biological replicates. P value were calculated using One-Way ANOVA with Fischer’s LSD test by comparing the mean of each activation and repression sample with the control activation sample where * = *P* ≤ 0.05, ** = *P* ≤ 0.01, *** = *P* ≤ 0.005, **** = *P* ≤ 0.0001, ns = *P* > 0.05.

Assuming no effect of unfused mTagBFP2, its DsRed2 MFI was set to zero. Subsequently, as expected, normalized MFI < 0 show repression, whereas MFI > 0 show activation of *P-HES1*. We then calculated the ratio of normalized MFI DsRed2 to the MFI of mTagBFP2-E6 including triple positive cells only. This ratio then resembles the specific activity of each E6 protein in this assay (Fig. 10) accounting for the different E6 expression levels (Fig. 9). A step to step analysis guide can be found in Supplementary information SI 5. As a result, the repressing activities of the beta-HPV 5E6 and 8E6 proteins are comparable, whereas 38E6 shows a 2-times and 4-times higher potential in repressing *P-HES1* regulated transcription of DsRed2 in H1299 and C33A cells, respectively. 16E6 shows a 5–7-times higher activation potential than the other E6 proteins of alpha-HPV 6bE6, 31E6, 18E6, which showed no significant impact on *P-HES1* activity in C33A cells. Surprisingly, a higher and more significant activation of the Notch pathway was observed in the p53-null H1299 cells for E6 of HPV 31 and 6b. Here the activity of 31E6 is even similar to the activity of 16E6. In principle, the trend of these semi-quantitative results is in line with the results above (Fig. 8). An additional accounting for the different E6 expression shows, that the individual activities of E6 proteins diverge largely in modulating the Notch pathway and are cell line dependent.

## Discussion

Notch signaling pathway plays an important role in cell fate determination and cell proliferation^1^. Dysregulation of Notch signaling is often observed in the progression of carcinoma^2,3^. Hence, it is extensively studied as a potential drug target to counter for treatment of its associated diseases. Numerous studies have shown the dysregulation of Notch signaling in HPV associated cancer progression by employing dual-luciferase assay^14–16^. However, the disadvantages of this luciferase reporter assay include the needs of the expensive commercial dual luciferase kit containing exogeneous substrates and cell lysis, disrupting the compartmentation of cells. The main limitation of luciferase assays is the comparison of the effect of different modulators on the reporter gene expression based on the expression of these modulatory proteins. To overcome these drawbacks, we established a triple fluorescence flow-cytometry-based reporter assay to analyze the effect of different HPV E6 proteins on Notch signaling pathway. The advantage of this flow-cytometry-based reporter assay is the triple gating strategy, which allows to monitor exclusively living cells that express the modulatory protein as well as the reporter and the activator. Reducing the background is especially advantageous if potential modulatory proteins show low transfection efficiencies and/or low steady-state expression levels. In addition to that, fluorescently labeled modulatory proteins allows quantification via flow-cytometry, possibly also in spatial localization which could give further insight into their modulating effect on gene regulation based on protein activity and protein amount.

Concerning the background of endogenous Notch signaling, which is tenfold lower than of the exogenously activated cells (Fig. 4). We expected that the E6 proteins also modulate the endogenous NICD. Indeed, we conducted experiments by co-transfecting C33A and H1299 cells with only *P-HES1*-DsRed2 reporter plasmid and mTagBFP2-E6 or mTagBFP2 (control) to examine the modulation effect of *P-HES1* activity by HPV16 and 8 E6 with endogenous NICD. In this case, a double fluorescence gating strategy was applied whereby the viable cells were gated for mTagBFP2 followed by DsRed2 (P1 > P1/mTagBFP2 > P1/mTagBFP2/DsRed2). Despite a similar overall trend, without heterologous expression of NICD, the % cells expressing DsRed2 in mTagBFP2-E6 cell population are too low to find a statistically relevant population to quantify differences (Supplementary information SI 6).

The effect of modulatory proteins on gene regulation can be influenced by protein activity (the more active the protein, the higher the effect) and the protein amount (the more protein, the higher the effect). In our assay, the expression level of mTagBFP2-E6 varies among the different HPV types. Different intracellular levels of E6 have been reported previously^21^ affecting the gene regulation by different E6 amounts. The expression levels of E6 are unknown in its native environment, during infection or cell transformation. Of course, the assay conditions do not fully resemble the native environment, especially with regard to the levels of E6, NICD and *P-HES1*. Nevertheless, and therefore allows a comparison of their activity this assay provides information about the specific activity of individual E6 proteins, which allow a comparison of each E6 protein potential in the context of dysregulating the Notch signaling pathway by HPVs. Our semi-quantitative analysis allows the comparison of different modulatory proteins, e.g. the different HPV E6 proteins, or mutants accounting for their individual variations in their expression levels. Overall, the triple fluorescence assay can be easily transferred to other genes of interest or signaling pathways by cloning the respective promotor, activator and modulatory protein into respective plasmids. Further it can be used to screen specific inhibitors of the modulatory proteins, here the HPV E6 proteins, and monitor their efficiency on the target gene expression in a fast way as well as in medium throughput.

Concerning the HPV E6 proteins and their biological functions, we could show that E6 proteins of different genus differentially manipulate the Notch signaling pathway. (I) all tested E6 proteins of beta-HPV repress the Notch pathway. This is in line with their reported interaction with MAML1 as a co-factor of the initiation complex of *HES1* transcription^2,12,14–16^. The repression potential is highest for 38E6, and lower for 5E6 and 8E6. 5E6 and 8E6 show a similar repression potential. HPV 38 belongs to beta-2 HPV, whereas HPV 5 and 8 belong to beta-1 HPV, indicating an association between function and phylogeny. (II) E6 of alpha-HPV do marginally interact with MAML1 and do not repress Notch signaling, as previously proposed for e.g. 16E6^12,14^. Indeed, the activity of the tested alpha-HPV E6 proteins is highly divergent. In C33A only 16E6 showed a clear activation. Higher levels of Notch signaling were previously reported for HPV16-positive keratinocytes and high-grade lesions ^19,20,22–24^. 18E6, 31E6 and 6bE6 showed no significant impact in C33A cells. (III) It is known, that E6 proteins of high-risk alpha-HPV 16, 18 and 31 lead to the degradation of the tumor suppressor p53^25–29^. Since there is a crosstalk between p53 and the Notch pathway^30,31^ we applied the same experiment in H1299 cells, which are p53 deficient. Here, the overall trend looks similar, beta-HPV E6 proteins repress and 16E6 activates the activity of the *P-HES1* reporter. Remarkably, the repressing effect in H1299 was so strong, that the triple positive cell population consisted of less than 500 cells. Nevertheless, the repressing effect of beta-HPV is clear and the tendency of the potentials of the different beta-HPV E6 proteins resembles the results measured in C33A. Whereas 18E6 again shows no effect on Notch signaling. H1299 31E6 and 6bE6 show a clear tendency to activate the Notch pathway in the p53-null cell line. Especially HPV 31E6, a high-risk type of the same species as 16E6, shows a strong activation similar to the activity of 16E6 and over two-fold stronger than the low-risk HPV 6bE6. Why 31E6 is more active in the p53-null cell line H1299 than in C33A, whereas the activity of the closely related 16E6 seems similar in both cell lines, is unknown. Both cell lines certainly differ in more than just the presence of p53. However, a speculative relation might be the lower potential of 31E6 interacting with^32^ and degrading p53^21^ than 16E6. E6 of HPV18, as the second most carcinogenic HPV type, surprisingly shows no significant effect on the Notch signaling in both cell lines. However, this approach focused only on the *P-HES1* promotor which is downstream of the Notch signaling pathway. To our knowledge, the effect of HPV18 on Notch signaling pathway has not been investigated so far. One speculation might be that HPV18 E6 is interfering with Notch signaling in a different way. Besides, to dysregulate cell proliferation many options exist despite Notch signaling.

It was previously reported that the HPV-related activation of the Notch pathway is driven by the transcription factor NFX123^22^, which is upregulated by 16E6. Presumably, the activation observed here depends on NICD downstream of gamma-secretase cleavage and impacts the formation of the transcription initiation complex (Fig. 1) directly or indirectly.

In conclusion, the triple fluorescence flow-cytometry-based assay is a novel suitable method to investigate the influence of exogenous proteins on the Notch pathway utilizing a Notch-responsive reporter plasmid. The possibility for semi-quantification allows a comparison of multiple modulatory proteins. Here, we utilized the assay to establish the differential regulation of the Notch-pathway by different HPV E6 proteins. In line with previous data, beta-1-HPV 5, 8 and beta-2-HPV 38 E6 proteins repress Notch, with 38E6 being the most potent repressor. Alpha-7 (16, 31) and alpha-10 (6b) HPV E6 proteins seem to activate the Notch pathway. Especially in the p53-null H1299 cell line both high-risk alpha-7 HPV 16E6 and 31E6 show a strong activation on *P-HES1 *activity. E6 of alpha-9 HPV18, which is the second most carcinogenic HPV after HPV16, had no effect on the Notch pathway. In summary the potency of the E6 proteins of HPV influencing the Notch pathway is highly variable even within the same genera but also between high-risk HPV types. Targeting the Notch pathway for HPV therapy requires an HPV-typing and individual adaption with respect to the HPV type.

## Materials and methods

### Plasmid DNA

Fluorescence based reporter plasmid *P-HES1*-DsRed2 (Addgene #13,767,^33^) and the Notch activation plasmid EF.ICN1.CMV.GFP (Addgene #17,623,^34^) were obtained from Addgene. HPV 16E6, 31E6, 18E6, 6bE6, 5E6, 8E6 and 38E6 (PAVE or GenBank protein reference numbers HPV16REF.1/GI:333,031, HPV31REF.1/GI:333,048, HPV18REF.1/GI:60,975, HPV6REF.1/GI:60,955, GenBank: CAA52689.1, HPV8REF.1/GI:333,074, HPV38REF.1/GI:1,020,234) constructs were cloned in pmTagBFP-C1 via restriction cloning or Gibson cloning obtaining an N-terminal Fusion of E6 with mTagBFP2.

### Cell culture

HPV negative cervical cancer cell line C33A and p53 null non-small cell lung carcinoma cell line H1299 were cultured in DMEM (Gibco, 41,965–062) supplemented with 10% fetal bovine serum (FBS) (Gibco, 10,270–106) and gentamicin (50 μg/mL) (Gibco, 157,710,049) at 37 degree Celsius, 95% humidity and 5% carbon dioxide. One day before transfection, 250 000 of C33A cells or 180 000 of H1299 cells were seeded in 12-wells sterile cell culture plate (Thermo Scientific, 150,628). Both cells were transfected with respective plasmid DNA using jetPRIME following manufacturer’s instructions on day 2. Control plasmid was always added to keep total amount of plasmid DNA transfected constant. Cells were trypsinized with Trypsin–EDTA (0.25%), phenol red (Gibco, 25,200,072) for FACS measurements or immunoblotting 48 h post-transfection for C33A cells or 36 h post-transfection for H1299 cells.

### FACS based reporter assay

For flo- cytometry, cells were harvested and washed in 500 µL precooled Dulbecco’s phosphate-buffered saline (DPBS) (Gibco, 14,190–169) containing 1% FBS. After washing, cells were resuspended in 200 µL precooled DPBS containing 1% FBS. FACS measurements were performed using MACSQuant VYB Flow Cytometer (Miltenyi Biotec) and analyzed with Flowlogic version 7.2.2 (Miltenyi–Inivai). Cells expressing the fluorescent proteins mTagBFP2, EGFP or DsRed2 were detected in channel V1 [405/450(50)] nm, B1 [488/450(50)] nm or channel Y2 [561/615(20)] nm, respectively. Three biological replicates were carried out for all experiments. For statistical analysis, we carried out Ordinary One-Way ANOVA with Fischer’s LSD test, comparing always all samples (E6) to the control (mTagBFP2) in Prism 9, version 9.1.2 (226). All figures presented in results and supplementary information were prepared using CorelDRAW X7, version 17.5.0.907. Example of the analysis method could be found in Supplementary information.

### Immunoblotting

Cells were lysed in lysis buffer [10% (v/v) Glycerol (MP Biomedicals, 4,800,689); 50 mM HEPES, pH 7.5 (Carl Roth, 9105.4); 3 mM Magnesium Chloride (Merck, 105,833); 0.1% (v/v) IGEPAL CA-630 (Merck, 18,896); 150 mM Sodium Chloride (Carl Roth, 3957.2); 1 mM Tris(2-carboxyethyl)phosphine (TCEP) (Alfa Aesar, J60316); 200 µM Zinc Chloride (Carl Roth, 3533); supplemented with Benzonase Endonuclease (Merck, 101,656), PhosSTOP (Roche, PHOSSRO) and cOmplete EDTA-free Protease Inhibitor Cocktail (Roche, COEDTAF-RO)]. The total protein concentration of the crude lysates was determined by Bradford assay and 70 µg total protein of the cell lysate were resolved on 8–20% SDS-PAGE gel. Proteins were electrotransfered onto nitrocellulose membrane (GE Healthcare) via wet blotting using blotting buffer (10 mM 3-(Cyclohexylamino)-1-propanesulfonic acid (CAPS) (Sigma, C2632), 0.001% (w/v) SDS (Carl Roth, 2326.2), 10% (v/v) methanol (Honeywell, 32,213), pH 10.3 at room temperature) for one hour at 70 V (constant). The membrane was blocked with 5% (w/v) Albumin Bovine Fraction V (Serva, 11,930) in phosphate buffer saline (PBS) one hour at room temperature and probed with appropriate primary antibodies overnight at 4 °C. Primary antibodies used at dilution of 1:1000 include anti-tRFP (Evrogen, AB233) for detection of mTagBFP2 and Living Colors A.v. Monoclonal Antibody (JL-8) (Takara Bio, 632,381) for detection of EGFP. DsRed2 was detected using Anti-DsRed (E-8) (sc-390909) and as a loading control HSP90 was detected by anti-HSP90 (4F10) (sc-69703) from Santa Cruz Biotechnology, both at a dilution of 1:200. Membrane was washed three times with PBS-T (0.05% v/v Tween 20). Secondary antibodies IRDye 680RD Goat anti-Rabbit IgG (H + L), IRDye 680RD Goat anti-Mouse IgG (H + L) or IRDye 800CW Goat anti-Mouse IgG (H + L) (LI-COR Biotechnology GmbH) were used at dilution of 1:10 000 and incubated for 30 min at room temperature. Membrane was again washed three times with PBS-T. The signal of respective protein was then visualized using LI-COR Odyssey Fc and analyzed with Image Studio Lite Software.

## Supplementary Information

Supplementary Information.
