# Supplementary material for: An enhanced triple fluorescence flow-cytometry-based assay shows differential activation of the Notch signaling pathway by human papillomavirus E6 proteins
Source: Sci Rep. 2022 Feb 22;12:3000. doi: 10.1038/s41598-022-06922-0 (PMC8863805; doi:10.1038/s41598-022-06922-0)
Supplement: Supplementary file 1 — Supplementary Information. [file 41598_2022_6922_MOESM1_ESM.pdf]

Supplementary Information

An enhanced triple fluorescence flow-cytometry-based assay shows differential activation of the Notch signaling pathway by Human Papillomavirus E6 proteins

JiaWen Lim[1], Elke Straub[1] Frank Stubenrauch[1] Thomas Iftner[1], Michael Schindler[1], Claudia Simon[1]*

Supplementary Information SI 1: Fluorescence microscopy

C33A and H1299 cells were grown on coverslip and transfected with 375 ng mTagBFP2 or 1500 ng of respective mTagBFP2-E6 and mounted on microscopy slide. Control plasmid was always added to keep total amount of plasmid DNA transfected constant. In order to have a clear differentiation between nucleus and cytoplasm, both cells were stained with HCS NuclearMask Red stain (Thermo Scientific) that for unknown reasons in our cell lines accumulated in the cytosol around the nucleus as evident by co-staining with DAPI (Thermo Scientific) (see Fig. SIa). Nevertheless, in co-staining with HCS NuclearMask Red stain it is evident that only mTagBFP2 is distributed all over the cells whereas mTagBFP2-E6 fusion is localized in the nucleus, except 6bE6 which is in the nucleus and cytosol (This is in line with literature data[35]). Please see Fig. SIb. All images are visualized with Zeiss Axio Observer Z1 Inverted Phase Contrast Fluorescence Microscope equipped with Apotome.2 under Plan-Apochromat 63x/1.40 oil immersion objective. Images were acquired using Axiocam 503 and Zen2 (blue edition), version 2.0.14283.302. Acquisition information and the dimension of the images are shown in Table SI2 and Table SI3.

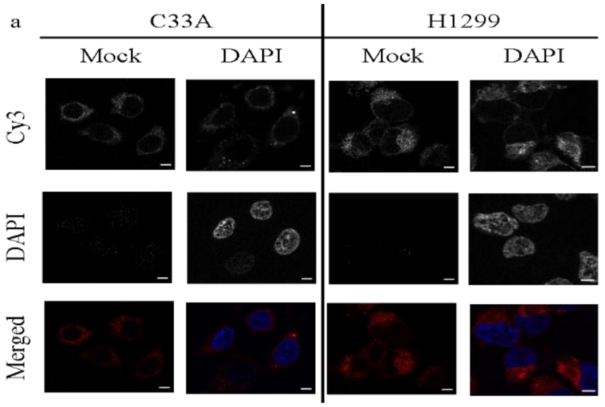

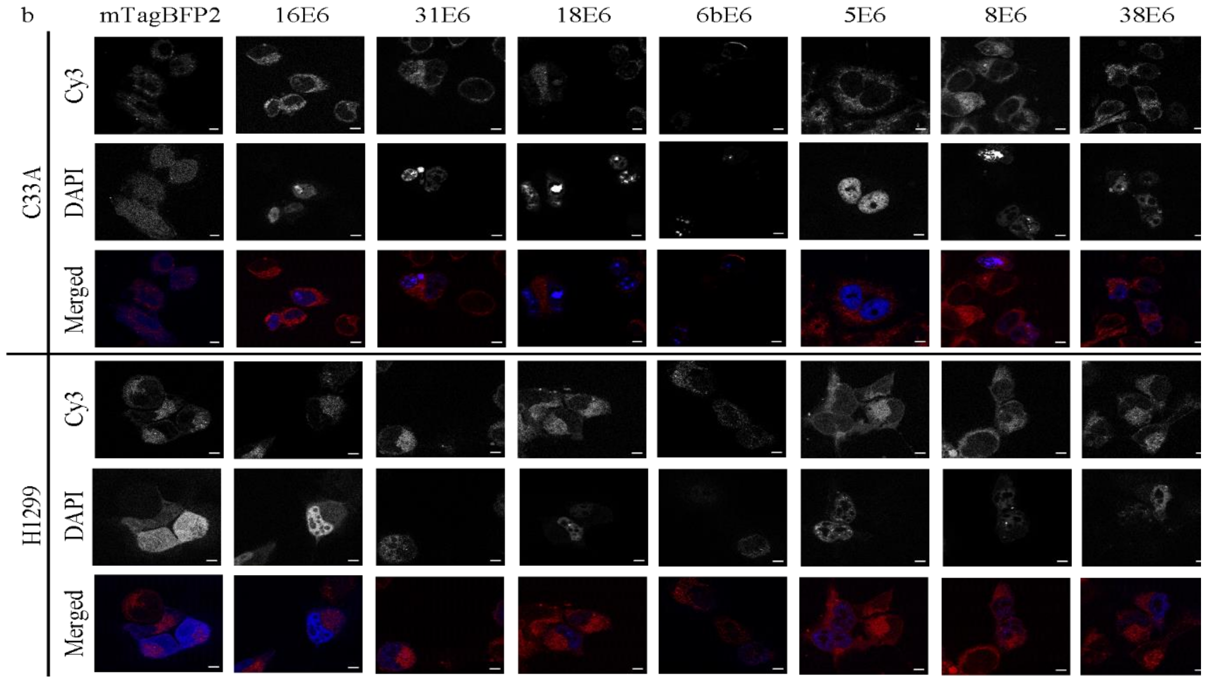

Figure SI1 Nuclear localization of mTagBFP2-E6.
C33A and H1299 cells were grown on coverslip and transfected with 375 ng mTagBFP2 or 1500 ng of respective mTagBFP2-E6. Control plasmid was always added to keep amount of total plasmid DNA transfected constant. After 48 hours post-transfection, cells were fixed with paraformaldehyde and nuclei were stained with HCS NuclearMask Red stain alone or co-stained with DAPI before mounted on microscopy slide. All images are visualized with Zeiss Axio Observer Z1 Inverted Phase Contrast Fluorescence Microscope equipped with Apotome.2 under 63x oil immersion objective. DAPI nuclear stained in a (exposure time = 15.3 ms) and mTagBFP2 or mTagBFP2-E6 in b (exposure time = 144.8 ms) were detected with DAPI channel while HCS NuclearMask Red stain was detected with Cy3 channel. a. HCS NuclearMask Red stain accumulates around the nucleus while DAPI shows a clear nucleus staining. b. mTagBFP2 alone localized everywhere in the cells while mTagBFP2-E6 localized in the nucleus. 18E6 and 6bE6 are expressed in the nucleoli whereas the other E6 are excluded from the nucleoli. 6bE6 localized in both nucleus and cytosol. All images were processed in Zen2 (blue edition) by cropped in the cells expressing mTagBFP2-E6 as shown. Scale bar = 5 µm.

Supplementary Information SI 2: Acquisition information of microscopy images

Table SI2 Acquisition information of microscopy images for C33A and H1299 cells.

Acquisition Information	HCS NuclearMask Red stain	mTagBFP2 / DAPI stain
Channel	Cy3	DAPI
Reflector	43 HE DsRed	49 DAPI
Beam splitter	570	395
Filter Ex. Wavelength	538-562	335-383
Filter Em. Wavelength	570-640	420-470

Supplementary Information SI 3: Image dimension of microscopy images

Table SI3 Image dimension of microscopy images during acquisition and export.

	C33A	H1299
Acquisition		
Image Size (Pixels)	968 x 728	968 x 728
Bit Depth	14 Bit	14 Bit
Exported		
Image size (Pixels)	448 x 448	455 x 425
Bit Depth	24 Bit	24 Bit

Supplementary Information SI 4: Full blot

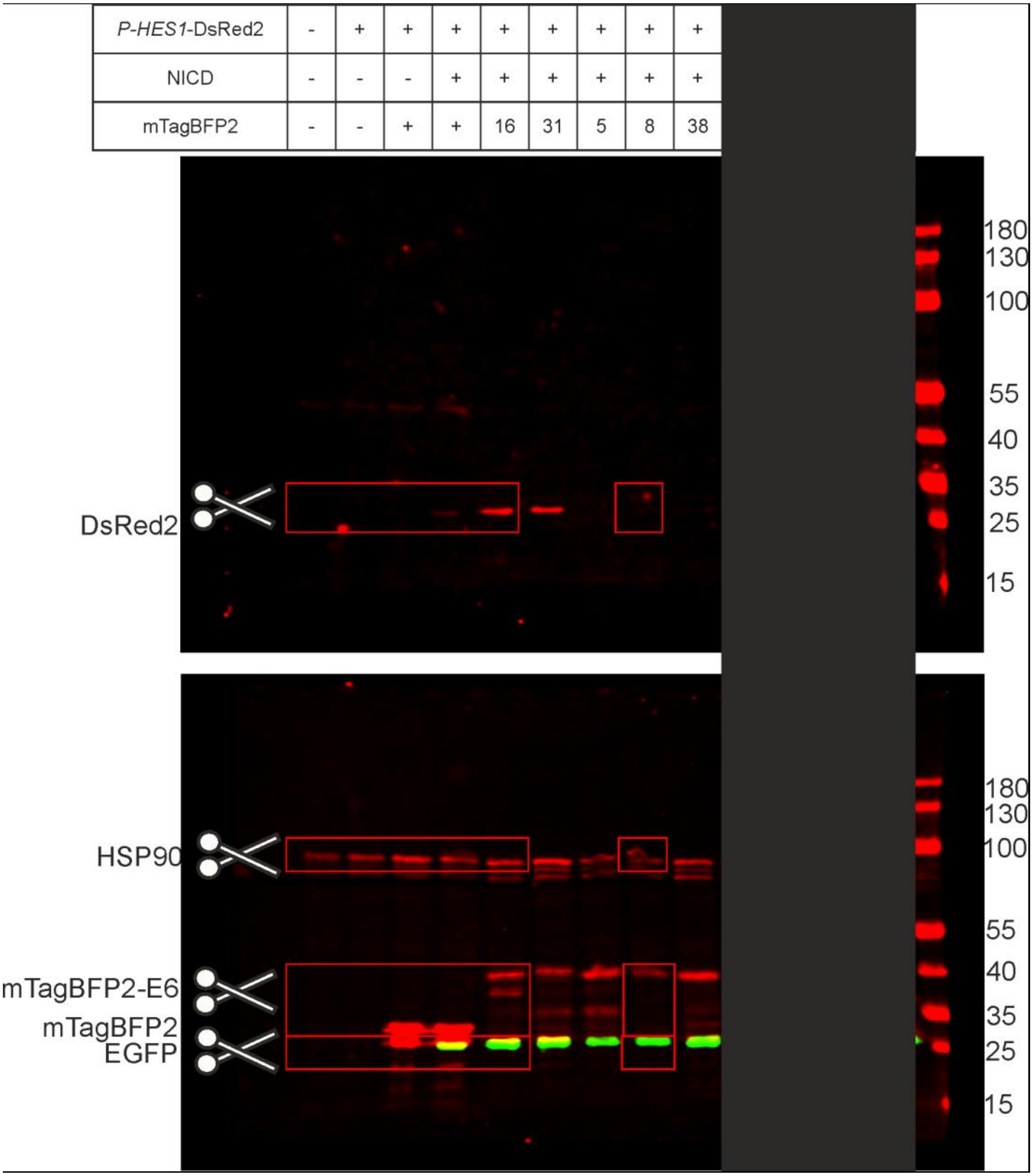

Figure SI4: Full length membrane blot of Figure 7 – Expression of proteins by Western blot analysis. Expression level on mTagBFP2 (27 kDa), mTagBFP2-E6 (~44kDa), DsRed2 (26kDa), and EGFP (27 kDa) were assessed by loading 70 µg total proteins on reducing 8-20% SDS-PAGE gel. HSP90 serves as a loading control. The blot was first incubated with anti-DsRed2 followed by IRDye 680RD Goat anti-Mouse (red). After visualization, the same blot was cut in between 55 kDa and 100 kDa marker band. The upper part of the blot was incubated with anti-HSP90 while the lower part was incubated with anti-tRFP to detect mTagBFP2

and mTagBFP2-E6. Then, upper part of the blot was incubated with IRDye 680RD Goat anti-Mouse (red) while the lower part with IRDye 680RD Goat anti-Rabbit (red). After visualization, the lower part of the same blot was incubated with Living Colors A.v. Monoclonal Antibody (JL-8) followed by IRDye 800RD Goat anti-Mouse (green) to detect EGFP. DsRed2 protein signal is increased for 16E6 but absent for 8E6. In line with FACS assay, these results indicate an activation by 16E6 and a repression for 8E6 of the Notch pathway. The cropped areas were highlighted with red boxes as shown above. Delineation of EGFP that shown in Figure 7 was cropped only in the green channel.

Supplementary Information SI 5: Guide to data analysis for triple fluorescence flow-cytometry-based assay

This supplementary information provides the step to step guide for the analysis of triple fluorescence flow-cytometry-based assay as shown in Fig. 8 and Fig. 10.

First, gate cells with the triple gating strategy as described in Fig. 6 to obtain data required for the following analysis.

➔ For the analysis shown in Fig. 8:

1. Import FACS data, either **% cells representing DsRed2 positive cell population** or **mean fluorescence intensity (MFI) that shows the intracellular expression level** of DsRed2 and mTagBFP2 from the **triple gated cells (P1/EGFP/mTagBFP2/DsRed2)**. Examples given below are calculated based on **<u>mean MFI of three independent biological replicates</u>**:

Table 1	Mean MFI P1/EGFP/mTagBFP2/DsRed2	
FPs	DsRed2	mTagBFP2
mTagBFP2	1239.6	40.7
8E6	632.2	57.7
16E6	1934.7	26.8

2. Calculate **relative promotor activity** as below:

$$\frac{\%\ cells\ or\ MFI\ of\ DsRed2\ obtained\ by\ modulator\ (E6)}{\%\ cells\ or\ MFI\ of\ DsRed2\ obtained\ by\ control\ (mTagBFP2)}$$

Table 2	*P-HES1* activity
mTagBFP2	1.0
8E6	0.5
16E6	1.6

➔ For the analysis shown in Fig. 10:

To calculate **Ratio of MFI of DsRed2: MFI of mTagBFP2**

First, set MFI of the control in to zero [called as delta DsRed2 (ΔDsRed2) from now on] by subtracting MFI of DsRed2 of modulator from Table 1 with MFI of DsRed2 of control from Table 1

MFI of DsRed2 of modulator (E6) – MFI of DsRed2 of control (mTagBFP2)

Table 3	ΔDsRed2
8E6	-607.5
16E6	695.1

Then, calculate Ratio of MFI of DsRed2 : MFI of mTagBFP2 as below:

$$\frac{\Delta DsRed2\ (from\ Table\ 3)}{MFI\ of\ mTagBFP2\ (from\ Table\ 1)}$$

Table 4	Ratio of MFI of DsRed2 : MFI of mTagBFP2
8E6	-11.1
16E6	25.7

Supplementary Information SI 6: Endogenous NICD is insufficient in activating *P-HES1* promotor

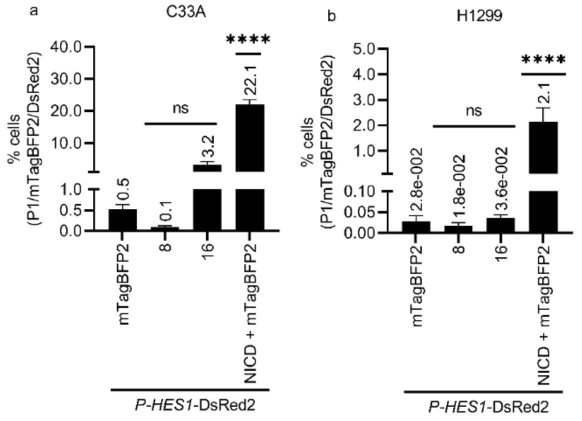

Figure SI6 DsRed2 positive cell population with/without exogenous NICD in C33A and H1299 cells. C33A and H1299 cells were transfected with 250 ng *P-HES1*-DsRed2, 125 ng mTagBFP2 or 500 ng mTagBFP2-E6, with or without 250 ng NICD as indicated. C33A cells were harvested 48 hours whereas H1299 cells were harvested 36 hours post transfection for FACS measurement. Living cells (P1) were double gated for mTagBFP2 followed by DsRed2 to examine the DsRed2 cell population activated with endogenous or exogenous NICD. A clear activation was observed in both cell line when NICD was co-transfected while endogenous NICD seemed to be insufficient to activate Notch activity. Co-transfection of modulator proteins mTagBFP2-E6 shows a similar trend as in mTagBFP2 without exogenous NICD. Three biological replicates were conducted in both cell lines with the mean value of % cells plotted and labelled above each bar. The error bars indicate the standard deviation of the mean value from the three independent replicates. P- values were calculated using One-Way ANOVA with Fischer's LSD test by comparing the mean each sample with mean of *P-HES1*-DsRed2 + mTagBFP2. **** = P≤0.0001, ns = P>0.05.
